# Complete Genome of Bacillus megaterium Podophage Pascal

**DOI:** 10.1128/genomeA.01429-14

**Published:** 2015-01-29

**Authors:** Jeffery D. Snowden, Alexander E. Vega Gonzalez, Justin W. Maroun, Adriana C. Hernandez, Gabriel F. Kuty Everett

**Affiliations:** Center for Phage Technology, Texas A&M University, College Station, Texas, USA

## Abstract

Podophage Pascal infects Bacillus megaterium, a commonly used model organism in biochemical research and an important industrial-scale protein production system. Here, we report the sequenced and annotated genome of Pascal and describe its prominent features. Bacteriophages such as Pascal may be valuable tools for research and industry.

## GENOME ANNOUNCEMENT

Bacillus megaterium was isolated in 1884 by Anton De Bary and is one of the most commonly known bacteria (1). It is a Gram-positive spore-forming bacterium. Its size and hardiness have led to its use as a model organism and as a protein production system for large-scale industrial operations (2). B. megaterium lytic bacteriophages can be detrimental to industrial applications, while temperate phages may be a valuable bioengineering tool in these fields. Phage Pascal is a novel podophage isolated against the asporogenic B. megaterium strain Km Sp.^-^.

Bacteriophage Pascal was isolated from a soil sample collected in College Station, TX. The phage DNA was sequenced in an Illumina MiSeq 250-bp paired-end run, with a 550-bp insert library, at the Genomic Sequencing and Analysis Facility at the University of Texas (Austin, TX). Quality-controlled trimmed reads were assembled to a single contig at 95.3-fold coverage using Velvet version 1.2.10. The contigs were confirmed by PCR to be complete. The genes were predicted using GeneMarkS (3) and corrected using the software tools available on the Center for Phage Technology (CPT) Galaxy instance (https://cpt.tamu.edu/galaxy-public/). Morphology was determined using transmission electron microscopy performed at the Texas A&M University Microscopy and Imaging Center.

Pascal has a 39,638-bp double-stranded DNA (dsDNA) genome with 50 predicted coding sequences, a G+C content of 39.9%, and a coding density of 96.3%. Twenty-five coding sequences have putative functions, as determined by BLASTp and InterProScan analyses (4, 5). Emboss Stretcher analysis shows that Pascal shares 67.2, 68.3, and 67.5% nucleotide sequence identities with B. megaterium podophages Pony (accession no. NC_022770), Page (accession no. NC_022764), and Pookie (accession no. KM236248), respectively (6). Pascal has a limited host range and infects B. megaterium strains Km Sp.^-^ and PV361.

Genes encoding the core proteins involved in morphogenesis (capsid, tail fiber, tail spike, and lytic tail protein), DNA replication (single-stranded DNA [ssDNA]-annealing protein, a DnaA-like replication initiator protein, and DnaB/D-like replication protein), transcriptional control (2 transcriptional repressors and an RNA polymerase sigma factor), lysis (holin, antiholin, and endolysin), and DNA packaging (small and large terminases and head-to-tail joining protein) were annotated. The large terminase is homologous to large terminases that use a *pac*-type headful packaging mechanism (7). As a *pac*-type phage, the circularly permuted genome of Pascal was opened to the small terminase gene by precedent (7). Of the two annotated transcriptional repressors, one contains a lambda Cro/CI-type DNA binding domain (IPR010982 and IPR001387) and the other contains an Arc-type DNA-binding domain (IPR013321) found in the transcriptional repressor of the temperate Salmonella phage P22 (8). The presence of these proteins may suggest a temperate lifestyle for phage Pascal.

An FtsK/SpoIIIE family protein is also encoded by the genome. These proteins are associated with intracellular DNA transfer in bacteria, as well as function in the sporulation in Bacillus by transferring DNA into the forespore (9). It is unknown how this family of proteins functions in bacteriophages, but we can speculate that they may be involved in the injection of phage DNA into the host.

### Nucleotide sequence accession number.

The genome sequence of phage Pascal was deposited in GenBank under the accession no. KM236247.
